# Supplementary material for: High-Fat Diet Augments Myocardial Inflammation and Cardiac Dysfunction in Arrhythmogenic Cardiomyopathy
Source: Nutrients. 2024 Jun 29;16(13):2087. doi: 10.3390/nu16132087 (PMC11243382; doi:10.3390/nu16132087)
Supplement: Supplementary file 1 [file nutrients-16-02087-s001.zip › nutrients-3076295-supplementary.pdf]

Supplementary Figures

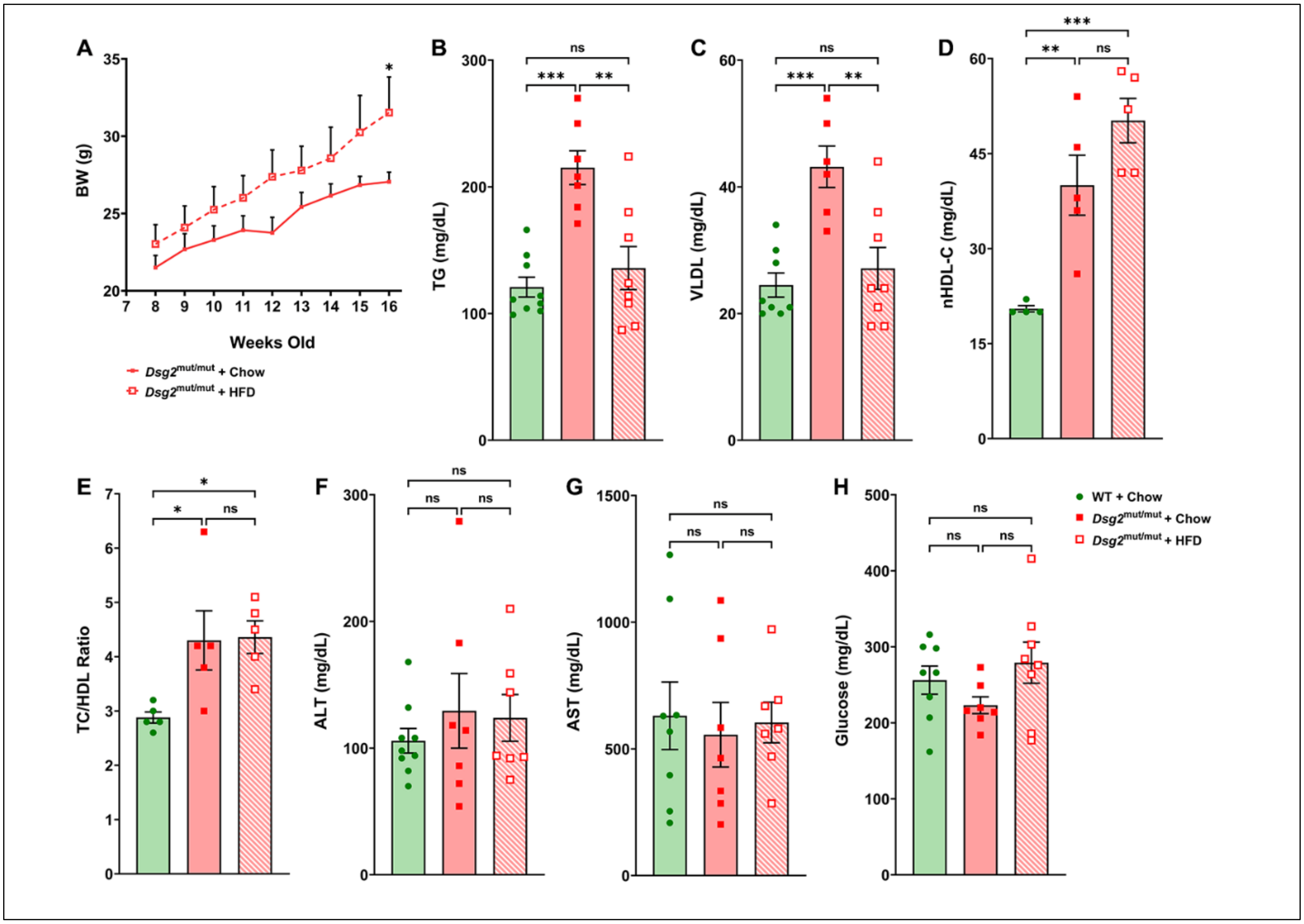

Figure S1. Weight gain and lipid perturbations in HFD-fed *Dsg2*$^{mut/mut}$ mice. (**A**) Body weight (BW) of mice was recorded weekly for the duration of the 8-week study between *Dsg2*$^{mut/mut}$ mice fed chow (*Dsg2*$^{mut/mut}$ + Chow) or HFD (*Dsg2*$^{mut/mut}$ + HFD). * $p \leq 0.05$ for *Dsg2*$^{mut/mut}$ + HFD vs *Dsg2*$^{mut/mut}$ + Chow at 16 weeks of age via two-way ANOVA with Tukey's posthoc analysis. Plasma markers were analyzed via Piccolo discs from 16-week-old mice for (**B**) triglycerides (TG); (**C**) very low-density lipoprotein (VLDL); (**D**) non-HDL cholesterol (nHDL-c); (**E**) the ratio of total cholesterol/high density lipoprotein (TC/HDL Ratio); (**F**) alanine aminotransferase (ALT); (**G**) aspartate aminotransferase (AST); and (**H**) glucose. Data presented as mean±SEM; n≥7 mice/cohort/parameter; ns, not significant; * $p < 0.05$, ** $p < 0.01$, *** $p < 0.001$, and **** $p < 0.0001$ via one-way ANOVA with Tukey's posthoc.

FOLD Change
<1
1-2
2-3
3-4
>4

Cytokine	*Dsg2* mut/mut (chow) vs WT (chow)		*Dsg2* mut/mut (HFD) vs WT (chow)		*Dsg2* mut/mut (HFD) vs *Dsg2* mut/mut (chow)	
	Fold Change	P-Value	Fold Change	P-Value	Fold Change	P-Value
Adiponectin	1-2	0.99	1-2	0.75	1-2	0.85
Amphiregulin	1-2	0.98	<1	0.96	<1	0.88
Angiopoietin-1	1-2	0.98	<1	0.94	<1	0.91
Angiopoietin-2	<1	1.00	1-2	0.94	1-2	0.93
Angiopoietin-like 3	1-2	1.00	1-2	0.99	1-2	1.00
BAFF/BLyS/TNFSF13B	<1	0.79	<1	0.99	1-2	0.86
C1q R1/CD93	<1	1.00	1-2	0.61	1-2	0.58
CCL2/JE/MCP-1	<1	0.97	1-2	0.98	1-2	0.91
CCL3/CCL4 MIP-1 a/B	<1	0.97	<1	1.00	1-2	0.97
CCL5/RANTES	1-2	0.98	1-2	0.78	1-2	0.89
CCL6/C10	1-2	0.98	1-2	0.97	1-2	1.00
CCL11/Eotaxin	1-2	0.85	<1	1.00	<1	0.81
CCL12/MCP-5	1-2	0.61	1-2	0.93	<1	0.83
CCL17/TARC	1-2	0.91	1-2	1.00	<1	0.93
CCL19/MIP-3B	<1	0.98	<1	0.97	<1	1.00
CCL20/MIP-3a	<1	0.73	<1	0.95	1-2	0.89
CCL21/6Ckine	<1	0.98	2-3	0.03*	3-4	0.02*
CCL22/MDC	<1	1.00	1-2	0.73	1-2	0.72
CD14	1-2	0.92	1-2	0.76	1-2	0.96
CD40/TNFRSF5	1-2	0.99	1-2	0.29	1-2	0.39
CD160	1-2	0.94	<1	0.92	<1	0.76
Chemerin	1-2	0.66	1-2	0.84	<1	0.94
Chitinase 3-like 1	1-2	0.41	2-3	0.24	1-2	0.95
Coagulation Factor III/ Tissue Factor	<1	1.00	1-2	0.86	1-2	0.86
Complement Component C5/C5a	<1	1.00	<1	1.00	1-2	1.00
Complement Factor D	<1	0.78	<1	0.95	1-2	0.92
C-Reactive Protein/CRP	<1	0.97	1-2	0.80	1-2	0.67
CX3CL1/Fractalkine	<1	0.98	2-3	0.03*	3-4	0.02*
KC (CXCL1)	1-2	0.88	1-2	0.97	<1	0.97
MIP-2 (CXCL2)	1-2	0.95	<1	0.98	<1	0.87
MIG (CXCL9)	1-2	0.99	<1	0.88	<1	0.82
IP-10 (CXCL10)	1-2	0.97	<1	0.87	<1	0.75
I-TAC (CXCL11)	1-2	0.35	<1	0.97	<1	0.24
CXCL13/BLC/BCA-1	2-3	0.21	1-2	1.00	<1	0.23
CXCL16	1-2	0.87	1-2	0.68	1-2	0.23
Cystatin C	1-2	1.00	1-2	0.94	1-2	0.96
DKK-1	<1	0.99	1-2	0.96	1-2	0.92
DPPIV/CD26	<1	0.87	1-2	0.92	1-2	0.64
EGF	1-2	1.00	1-2	0.96	1-2	0.97
Endoglin/CD105	1-2	1.00	1-2	0.44	1-2	0.49
Endostatin	<1	0.96	1-2	0.52	2-3	0.38
Fetuin A/AHSG	1-2	0.99	1-2	0.87	1-2	0.94
FGF acidic	1-2	1.00	1-2	0.97	1-2	0.98
FGF-21	1-2	0.99	<1	0.84	<1	0.80
Flt-3 Ligand	1-2	0.81	<1	1.00	<1	0.81
Gas 6	1-2	0.59	<1	1.00	<1	0.51
G-CSF	1-2	0.73	<1	0.83	<1	0.39
GDF-15	<1	0.99	<1	0.91	<1	0.96
GM-CSF	<1	0.89	<1	0.96	1-2	0.98
HGF	<1	0.95	1-2	0.93	1-2	0.79
sICAM-1 (CD54)	<1	0.96	1-2	0.48	2-3	0.35
IFN-y	1-2	0.84	1-2	1.00	<1	0.88
IGFBP-1	1-2	1.00	1-2	0.73	1-2	0.80
IGFBP-2	<1	0.84	1-2	0.93	1-2	0.63
IGFBP-3	<1	1.00	<1	0.85	<1	0.88
IGFBP-5	1-2	0.95	<1	0.99	<1	0.90
IGFBP-6	1-2	0.69	2-3	0.21	1-2	0.70
IL-1a (IL-1F1)	2-3	0.22	<1	1.00	<1	0.19
IL-1B (IL-1F2)	1-2	0.47	<1	1.00	<1	0.46
IL-1ra (IL-1F3)	1-2	0.88	1-2	0.96	<1	0.97
IL-2	1-2	1.00	1-2	1.00	1-2	1.00
IL-3	1-2	0.88	1-2	1.00	<1	0.88
IL-4	1-2	0.90	1-2	0.97	<1	0.98
IL-5	1-2	0.41	1-2	0.92	<1	0.64
IL-6	1-2	0.77	1-2	0.75	1-2	1.00
IL-7	<1	0.96	1-2	0.74	1-2	0.59
IL-10	1-2	0.90	<1	0.93	<1	0.71
IL-11	1-2	0.79	<1	0.93	<1	0.57
IL-12p40	2-3	0.15	1-2	1.00	<1	0.18
IL-13	2-3	0.24	1-2	0.99	<1	0.31
IL-15	1-2	0.54	1-2	0.91	<1	0.78
IL-17A	2-3	0.08	1-2	0.86	<1	0.22
IL-22	<1	0.91	1-2	0.82	1-2	0.58
IL-23	1-2	1.00	1-2	0.90	1-2	0.94
IL-27p28	1-2	0.90	1-2	0.74	1-2	0.96
IL-28A/B	1-2	0.56	1-2	0.72	<1	0.96
IL-33	<1	0.95	1-2	0.62	2-3	0.44
LDLR	<1	0.87	1-2	0.68	2-3	0.39
Leptin	1-2	0.75	<1	1.00	<1	0.74
LIF	1-2	0.44	1-2	0.98	<1	0.57
Lipocalin-2/NGAL	2-3	0.07	2-3	0.01*	1-2	0.88
LIX	2-3	0.22	1-2	0.76	<1	0.59
M-CSF	1-2	0.90	1-2	0.69	1-2	0.94
MMP-2	1-2	0.97	2-3	0.02*	2-3	0.06
MMP-3	<1	1.00	3-4	<0.0001*	3-4	<0.0001*
MMP-9	<1	0.79	3-4	0.003*	>4	0.0003*
Myeloperoxidase/MPO	1-2	1.00	2-3	0.02*	2-3	0.03*
Osteopontin (OPN)/Spp1	1-2	0.99	2-3	0.05*	2-3	0.09
Osteoprotegerin/TNFRSF11B	<1	0.88	1-2	0.85	2-3	0.57
PD-EGF/ Thymidine phosphorylase	<1	0.76	1-2	0.99	2-3	0.69
PDGF-BB	1-2	1.00	1-2	0.92	1-2	0.96
Pentraxin 2/SAP	3-4	0.005*	2-3	0.02*	<1	0.89
Pentraxin 3/TSG-14	2-3	0.03*	1-2	0.47	<1	0.32
Periostin/OSF-2	2-3	0.03*	2-3	0.14	<1	0.74
Pref-1/DLK-1/FA1	1-2	0.51	1-2	0.83	<1	0.84
Proliferin	1-2	0.99	1-2	0.53	1-2	0.64
Proprotein Convertase 9/PCSK9	1-2	0.67	2-3	0.13	1-2	0.55
RAGE	1-2	0.68	1-2	0.29	1-2	0.82
RBP4	1-2	1.00	1-2	0.84	1-2	0.90
Reg3G	1-2	0.84	1-2	0.99	<1	0.89
Resistin/Retn	1-2	1.00	2-3	0.04*	2-3	0.06
E-Selectin/CD62E	1-2	0.77	1-2	0.75	1-2	1.00
P-Selectin/CD62P	2-3	0.26	2-3	0.17	1-2	0.99
Serpine 1/PAI-1	2-3	0.27	3-4	0.0002*	1-2	0.06
Serpin F1/PEDF	2-3	0.16	1-2	0.27	<1	0.94
Thrombopoietin	1-2	0.39	1-2	0.93	<1	0.60
TIM-1/KIM-1/HAVCR	2-3	0.13	1-2	0.68	<1	0.50
TNF-alpha	3-4	0.004*	1-2	0.54	<1	0.07
VCAM-1/CD106	1-2	0.68	1-2	0.39	1-2	0.91
VEGF	1-2	0.52	1-2	0.97	<1	0.67
WISP-1/CCN4	1-2	0.53	1-2	0.57	<1	0.99

Figure S2. Increased myocardial inflammatory cytokines from *Dsg2*$^{mut/mut}$ mice fed a HFD. Fold-change and *p*-values, assessed via one-way ANOVA with Tukey's posthoc analysis from 16-week-old *Dsg2*$^{mut/mut}$ mice fed chow or HFD (*Dsg2*$^{mut/mut}$ + Chow and *Dsg2*$^{mut/mut}$ + HFD; respectively) and WT mice fed chow (WT + Chow).

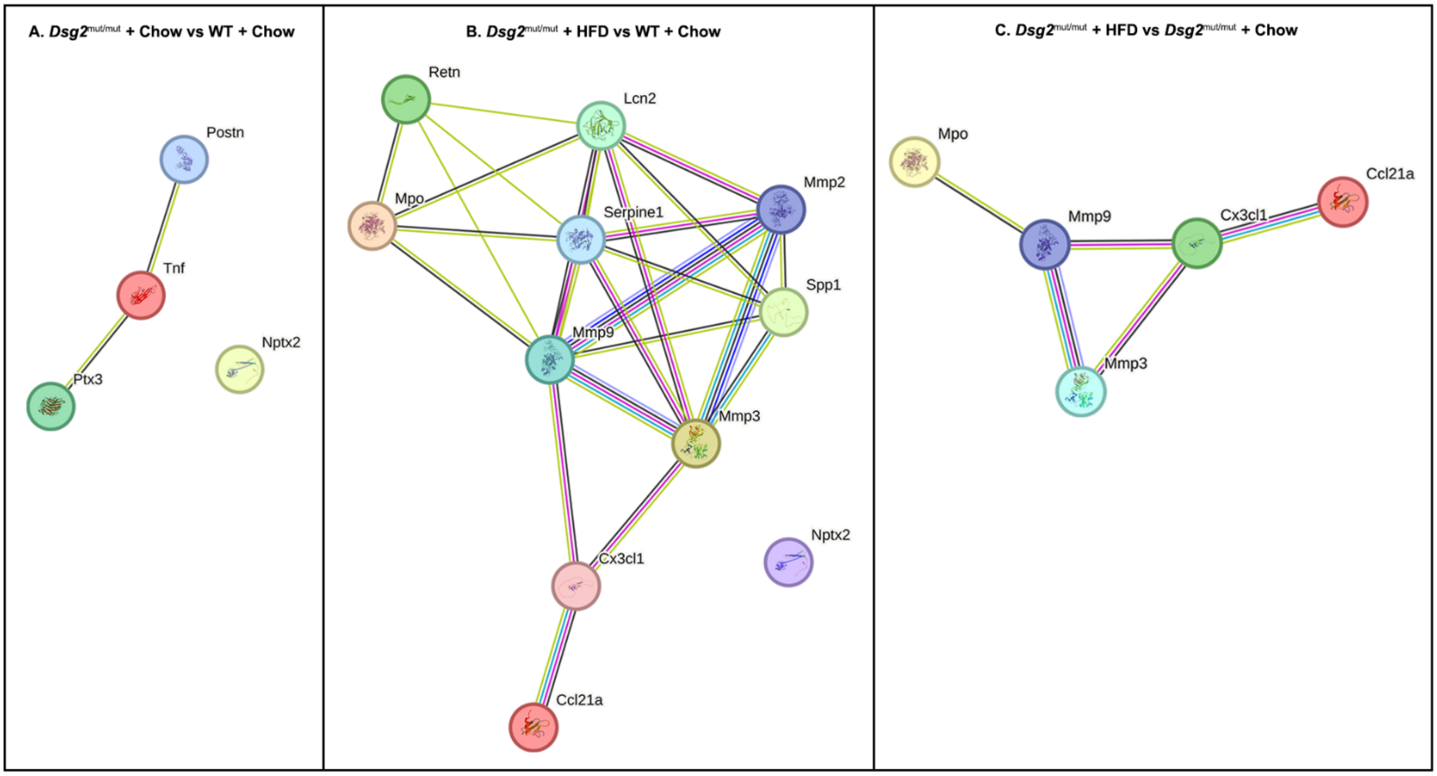

Figure S3. Associations between cardiac inflammatory markers in *Dsg2*$^{mut/mut}$ mice via STRING analysis. (**A-C**) Predicted protein-protein interactions from 16-week-old mice via STRING analyses from cytokines that were significantly upregulated in (**A**) *Dsg2*$^{mut/mut}$ mice fed chow (*Dsg2*$^{mut/mut}$ + Chow) vs WT mice fed chow (WT + Chow); (**B**) *Dsg2*$^{mut/mut}$ mice fed HFD (*Dsg2*$^{mut/mut}$ + HFD) vs WT + Chow; and (**C**) *Dsg2*$^{mut/mut}$ + HFD vs *Dsg2*$^{mut/mut}$ + Chow. Periostin, Postn; tumor necrosis factor alpha, Tnfα; pentraxin-3, Ptx3; pentraxin-2, Nptx2; matrix metalloproteinase-2, -3, and -9, Mmp-2, Mmp-3, and Mmp-9, respectively; osteopontin, Spp1; lipocalin-2, Lcn2; myeloperoxidase, MPO; Resistin, Retn; C-X3-C motif chemokine ligand-1, Cx3cl1; and C-C motif chemokine ligand-21, Ccl21a.
